# Second Harmonic Generation Nanoparticles for Biomedical Imaging: Synthesis and Interaction with Model Bio-Interfaces

**DOI:** 10.3390/molecules31030416

**Published:** 2026-01-26

**Authors:** Irene Nepita, Maria Teresa Buscaglia, Belen Arcos-Álvarez, Eduardo Guzmán, Eva Santini, Libero Liggieri, Francesca Ravera

**Affiliations:** 1 CNR-Institute of Condensed Matter Chemistry and Technology for Energy, Unit of Genoa, 16149 Genoa, Italy; irene.nepita@gmail.com (I.N.); mariateresa.buscaglia@cnr.it (M.T.B.); eva.santini@cnr.it (E.S.); libero.liggieri@cnr.it (L.L.); 2 Departamento Química Física, Facultad Ciencias Químicas, Universidad Complutense Madrid, 28040 Madrid, Spain; belenarc@ucm.es (B.A.-Á.); eduardogs@quim.ucm.es (E.G.); 3 Instituto Pluridisciplinar, Universidad Complutense, 28040 Madrid, Spain

**Keywords:** barium titanate, biomedical imaging, nanoparticles, second harmonic generation, tensiometry

## Abstract

This work investigates the synthesis and characterization of Barium Titanate (BT) nanoparticles, which exhibit non-linear optical properties, with a focus on their potential application in biomedical imaging. BT nanoparticles are active in second harmonic generation (SHG), enabling deep tissue imaging with a high signal-to-noise ratio. A major objective of this study is to advance in the understanding of the interactions between these nanoparticles and model biological systems. To this end, monolayers of 1,2-dipalmitoyl-sn-glycerol-3-phosphocholine (DPPC) spread on aqueous sub-phase are employed as model bio-interfaces, and the effects of BT nanoparticles on their properties are investigated using physicochemical experimental techniques such as Langmuir trough and Dynamic Light Scattering, also in the presence of albumin, a representative serum protein. The results evidence nanoparticle incorporation into the lipid layer, affecting its phase behavior, as well as the spontaneous formation of protein coronas around NPs, which is further confirmed by super-resolution optical microscopy

## 1. Introduction

Nanoparticles (NPs) with nonlinear optical properties, such as those based on second harmonic generation (SHG), are very promising for the development of innovative nano-probes in the field of optical diagnostics [1,2,3] ensuring high signal-to-noise ratio and deep non-invasive tissue imaging employing near-infrared light. However, challenges persist regarding SHG nano-probes’ interaction with biological membranes and fluids, which may impact their diagnostic performance and induce potential adverse effects [1,4]. Important aspects to be investigated concern, for example, the conditions for the incorporation of these nanomaterials into biological membranes and their possible effects on the structure, composition and rheological properties of such membranes [5]. Another critical issue is the adsorption and accumulation of blood serum proteins onto nanoparticle surface, which may block the in-vivo targeting of nanomaterials and promote cytotoxicity [6]. In addition, the formation of bio-molecular corona [7] may become crucial when these materials are employed as in vitro diagnostic sensors, in vivo diagnostic probes, or for drug delivery technologies [8]. In this context, investigating SHG NPs, from their synthesis and physicochemical characterization to their interaction with model bio-systems, is especially relevant for their utilization in the development of efficient non-invasive nano-probes for optical diagnostic-based bio-imaging.

This work focuses on the synthesis and characterization of Barium Titanate (BT) powders. Among the materials exhibiting second harmonic generation properties, BT stands out as a prototypical ferroelectric perovskite oxide with high dielectric permittivity and piezoelectric response [9,10]. Thanks to its large dielectric constant, spontaneous polarization, and strong electro-optical response, BT has long been employed in a wide range of photonic and optoelectronic applications, including ultrafast image processing, memory and storage systems, and advanced imaging technologies [11]. Recent studies have highlighted the versatility of BT in emerging areas such as photocatalysis and advanced optical and nonlinear-optical applications, demonstrating renewed relevance through doped systems and transparent composites with unconventional dielectric and optical responses at room temperature [12,13].

Beyond these technological fields, BT has also attracted considerable interest in biomedicine, where it has proven to be a promising material for the development of nano-probes for biological detection and diagnostics, as well as for site-specific drug delivery applications [14].

General aim of this work is to advance in the understanding of the interaction mechanisms of BT micro- and nanoparticles with biomolecules and/or lipid layers that mimic realistic diagnostic environments.

In previous studies [15,16,17], it has been shown that important insights into the effect of NPs of various morphologies and chemical compositions on the structure and properties of biologically relevant systems can be obtained by investigating their interaction with Langmuir monolayers of fatty amphiphiles at the water/air interface. These monolayers can, in fact, be assumed as simplified yet informative models of the real bio-interface.

These approaches rely on the well consolidated concept that solid nanoparticles, once incorporated into adsorption/spread layers, can modify their thermodynamic and rheological behaviours, thereby affecting interfacial structure and composition [15,16,17]. Such effects can be quantitatively evaluated through interfacial tension measurements.

In this work, BT particles are synthesized using an optimized hydrothermal-like approach that ensures controlled particle size and improved colloidal stability. Their interaction with Langmuir monolayers of 1,2-dipalmitoyl-sn-glycerol-3-phosphocholine (DPPC), as well as the behavior of these particles in the presence of albumin, are investigated using physicochemical methods and optical microscopy. The choice of DPPC is motivated by the fact that it is the main component of relevant bio-interfaces, such as the cell membrane or lipid matrix of pulmonary surfactant, while albumin has been selected because it is the most abundant protein in human serum.

## 2. Results and Discussion

### 2.1. Properties of BT Particles

**Figure 1:**
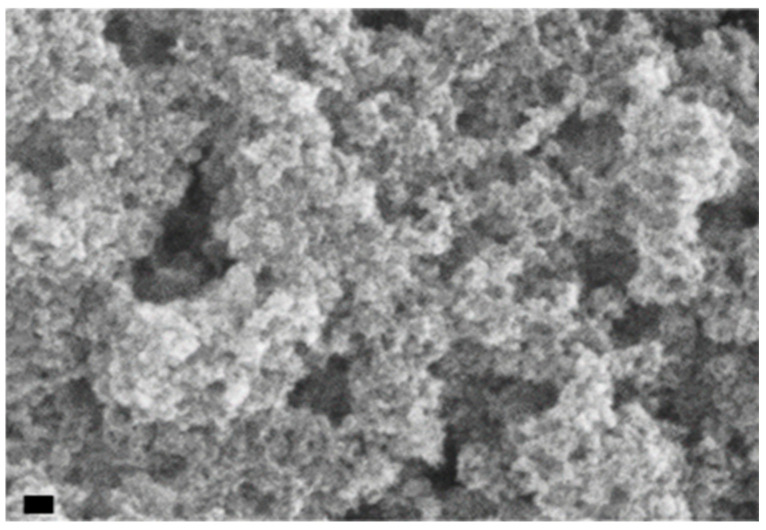
SEM image of synthesized dried BT particles. Scale bar 100 nm.

The method described in detail in Section 3, allows ultrafine BT NPs to be synthesized. As shown in previous works [18,19], depending on the processing parameters, such as temperature and cation concentration, the final particle diameter can be tailored from 10–20 nm to more than 1 μm. More specifically, the protocol adopted in the present study provides particles with an average size of 15–20 nm which correspond to a specific surface area of 75 m^2^/g, confirmed by SEM analysis (Figure 1) and BET surface measurement [18].

For the aims of the present work, aqueous dispersions of BT particles are prepared and characterized according to the procedure described in Section 3. An appropriate colloidal stability of these dispersions is obtained by the addition of polyacrylic acid (PAA), a commonly used and highly effective dispersant for this class of nanomaterials [19,20]. The concentration of PAA was optimized in order to obtain an appropriate balance between the stabilizing effect and the minimum modification of the composition of the aqueous phase. To this purpose, dispersions with different concentrations of PAA and given content of BT particles were analyzed by optical microscopy for a first evaluation and then centrifuged in order to separate and characterize the nanosized content of the dispersion by Dynamic Light Scattering. By this study, it was possible to conclude that the best dispersions, as concerns stability and size distribution, are obtained with a ratio between the dispersing agent and particle concentrations of 2.45 × 10^−5^ moles of PAA per gram of BT particles. This ratio corresponds to a particle surface coverage around 0.8, estimated assuming the BT specific surface area measured for the synthesized particulate and the namely value of the PAA molecular area. For all the BT dispersions investigated in this work, whether or not specified, an amount of PAA was added, in order to have a PAA/BT concentration ratio in agreement with this evaluation.

**Figure 2:**
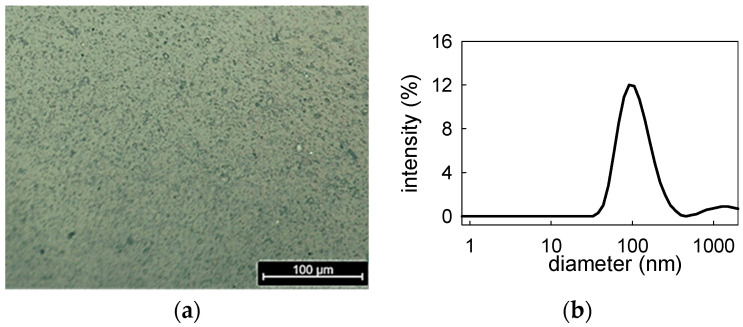
Image by optical microscopy of BT dispersion at 0.2 g/L, stabilized by PAA at 4.9 × 10^−6^ M (**a**), and intensity-weighted particle size distribution by DLS measurements of the supernatant obtained by the centrifugation of the same dispersion at BT concentration of 0.02 g/L (**b**).

BT particle dispersions at 0.2 g/L and PAA concentration of 4.90 × 10^−6^ M were specially produced and investigated. Such dispersions present a good stability over time. Moreover, the inspection by optical microscopy evidences the presence of particles with sizes in the range of 1–10 µm which are expected to be aggregates of primary NPs (Figure 2a). To increase the particle size information on a larger scale, the dispersions have been centrifuged at 5.500 rpm for 10 min and the obtained supernatant analyzed by DLS and zeta potential measurements. The size distribution obtained presents a maximum around 100 nm (Figure 2b) and the measurement of ζ-potential provided −11.2 ± 0.9 mV, at neutral pH. Despite the rather low value of the ζ-potential and the fact that the PAA component, at neutral pH, can be assumed only weakly dissociated, the obtained nanoparticle dispersions present a very good long-term stability. In fact, size distributions in excellent agreement with that reported in Figure 2b, i.e., average diameters within 96–103 nm, have been obtained by DLS measurements of the same dispersions in a time range of the order of months.

To obtain the concentration of the supernatant dispersion a simple procedure is adopted, which consists in separating the lower part of the dispersion containing the sedimented micrometric powder, measuring its weight after drying and, from this value, calculating the content in NPs of the left supernatant. In this specific case, the concentration of the BT NPs dispersion has found to be 0.02 g/L.

**Figure 3:**
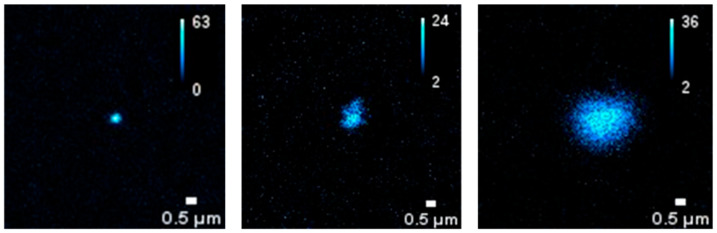
SHG imaging of BT dispersion obtained using the optical microscope described in Section 3, with an excitation wavelength of 1150 nm and a power of 100 mW.

For these dispersions, the SHG properties of the BT nanoparticle assemblies were assessed using the detection device described in detail in Ref. [21]. The images reported in Figure 3 confirm that BT particles exhibit appreciable second-harmonic generation under near-infrared excitation (λ_exc_ = 1150 nm, P = 100 mW). Notably, a detectable SHG signal was observed from PAA-stabilized BT assemblies in the 1–5 µm size range. This observation adds further evidence that SHG detectability, previously demonstrated mainly in the sub-micrometric regime (including single nanoparticles down to ~22 nm) [18], can be extended to micrometer-scale structures that are directly relevant for biomedical imaging applications.

### 2.2. BT NPs Interaction with DPPC Monolayers

The interaction between BT NPs and DPPC monolayers has been investigated by employing the Langmuir trough technique (see Section 3), following a protocol similar to that reported in ref. [17].

#### 2.2.1. Effect of Temperature

Investigating the compression isotherm, or surface pressure, Π, versus the area per molecule, of Langmuir monolayers of DPPC spread onto aqueous dispersions of BT NPs is an effective way to get insights into the mechanisms of NPs–DPPC interaction. In particular, the dependence on temperature of these properties is very important for the potential application in bio-imaging.

**Figure 4:**
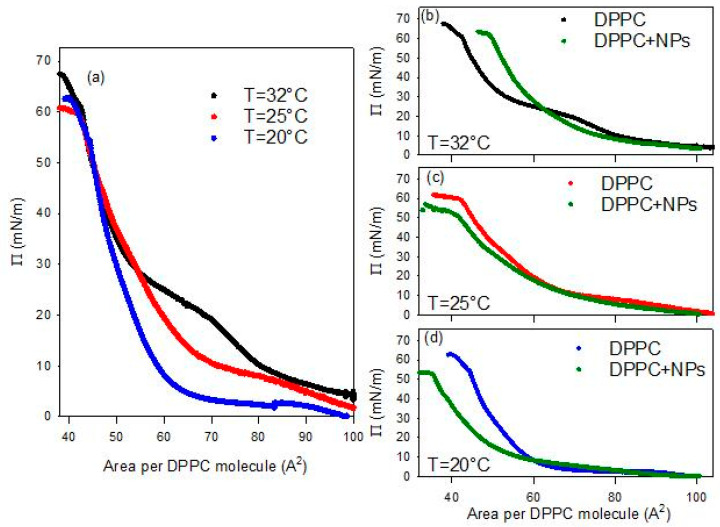
Surface pressure-area per DPPC molecule isotherms for DPPC monolayers spread at the air/water interface at different temperatures (**a**) and compared with that obtained for DPPC monolayers spread onto a 0.01 g/L dispersion of BT NPs at 32 °C (**b**), 25 °C (**c**) and 20 °C (**d**). The reported isotherms are the average isotherm obtained from 3 independent measurements with a deviation below 5%.

As first stage of this study, the compression isotherm of DPPC spread at the bare water/air interface was evaluated at three different temperatures (in the range 20–32 °C). The temperature range was strategically selected to span ambient environmental conditions up to near-physiological temperatures relevant for deep-tissue SHG imaging applications (~37 °C). The results are reported in Figure 4a. A further effective way to analyse the changes in the phase behavior of the spread monolayers and put them in relation with their mechanical properties, is to calculate the quasi-equilibrium dilational elasticity, *E*_0_, defined as the variation of the surface pressure due to the relative area reduction, that is

(1) $$E_{0}=-A{\left(\frac{\partial \Pi }{\partial A}\right)}_{T}.$$

**Figure 5:**
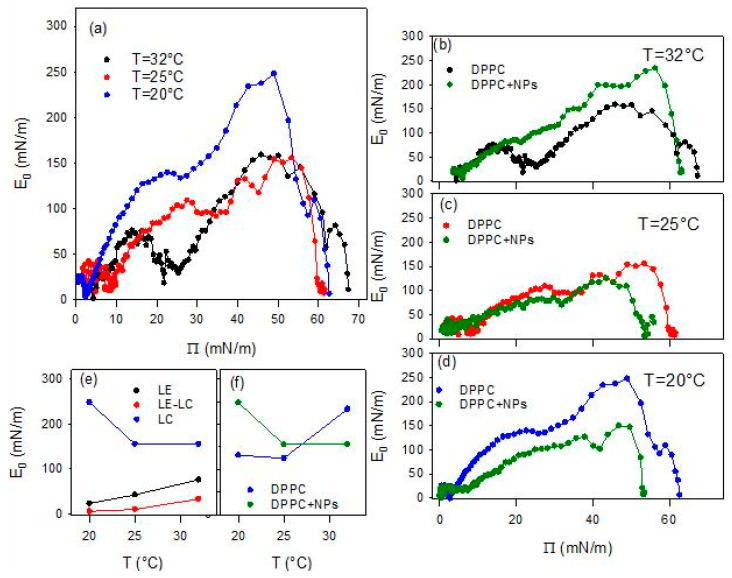
Quasi-equilibrium dilational elasticity, E_0_, versus surface pressure, Π, for DPPC monolayers spread at the air/water interface at different temperatures (**a**), and the same compared with E0 obtained for DPPC monolayers spread onto a dispersion of BT particles of concentration 0.01 g/L at 32 °C (**b**), 25 °C (**c**) and 20 °C (**d**). Temperature dependence of the relative maximum values (LE and LC phases) and minimum value (LE-LC coexistence phase) of E_0_ (**e**), and of the maximum E_0_ values found for DPPC at air/water and air/dispersion interfaces (**f**).

$E_{0}$ was calculated numerically from the experimental Π–A isotherms. Each isotherm was first resampled to 100 equally spaced in molecular area data points using the interpolation tool of OriginPro 9.0 (OriginLab Corporation, Northampton, MA, USA), in order to minimize experimental noise while preserving the shape of the curve. The derivative ∂Π/∂A was then obtained by central finite differences on the resampled data, and the corresponding $E_{0}$ values were computed for each point. Test calculations performed with higher resampling densities and smoothing levels increase the noise, which evidences that the correct choice of the numerical procedures is critical. In Figure 5 the quasi-equilibrium elasticity values calculated from the data of Figure 4 are plotted versus the surface pressure.

At a first analysis of the isotherms reported in Figure 4a, it is evident that the surface pressure evolution during the monolayer compression presents similar features regardless of temperature. In agreement with literature [22], at the highest values of the area per DPPC molecule the surface pressure increases progressively passing through gas and liquid expanded (LE) phases, until reaching a compression degree where the surface pressure remains almost constant with further compression. This quasi-plateau corresponds to the coexistence between a LE and a liquid condensed (LC). As the compression advances in this region, the LC phase is formed at the expense of the LE one, under the effect of the increasing ordering of the lipid molecules in the interfacial layer. Once the coexistence region is overcome, only the LC phase exists and a sharper increase of the surface pressure under compression is observed, resulting from the poor compressibility of such phase. When the ordering of lipids is nearly complete, the interface behaves like a 2D solid and the monolayer collapses at a surface pressure above 60 mN/m.

Despite the similarities in the general evolution, a clear temperature dependence of the main features of the compression isotherm is observed. In particular, this temperature-dependent behavior affects the LE-LC coexistence region (quasi-plateau). In fact, increases in temperature cause the shift of the DPPC isotherm toward larger areas per molecule, or equivalently, the increase in surface pressures at a given area. The plateau becomes less pronounced and is displaced to higher pressures in agreement with the experimental results by Crane et al. [23] and the simulations by Duncan and Larsson [24]. The observed changes may be ascribed to enhanced thermal motion of the lipid acyl chains at elevated temperatures, which results in increased surface pressure [25]. Moreover, in agreement with literature [26,27], the obtained results show that the DPPC isotherms at different temperatures vary within the coexistence region but converge at both extremes, i.e., at the highest and lowest values of area available per DPPC molecule. This is confirmed by the negligible change with temperature of the limit area (A_lim_), defined as the minimum area per molecule before the collapse. The measured values of this parameter have been found to be about 40 A^2^/molecule for all temperatures.

The above scenario is confirmed by the temperature dependence of the quasi-equilibrium dilational elasticity (Figure 5). In fact, the change in the phase behavior associated with the increase of the temperature is evidenced by the average reduction of E_0_ for DPPC monolayer, especially that corresponding to the most ordered phase (LC phase). This is clearer from the values reported in Figure 5e where the maximum values of the quasi-equilibrium elasticity within the LE and LC phases, and the minimum value within the LE-LC coexistence are reported. On the other hand, the fact that the presence of a pseudo-plateau tends to disappear as the temperature increases is reflected in the value of E_0_ within this region, which passes from an almost vanishing value, lower than 5 mN/m (close to the null value expected for a first order phase transition), at 20 °C, to an appreciable value around 20mN/m, at 32 °C.

The behavior of DPPC monolayer changes markedly when the lipid is spread onto an aqueous dispersion containing BT NPs (see Figure 4b–d). It is worth noting that all experiments deal with PAA-coated BT nanoparticles (PAA/BT = 2.45 × 10^−5^ mol/g, surface coverage θ ≈ 0.8), where PAA functions as the optimized stabilizer ensuring dispersion stability while enabling nanoparticle-DPPC interactions. The observed alteration may be ascribed to the incorporation of particles into the DPPC monolayers, likely mediated by weak electrostatic interactions between the negatively charged groups on the surface of the particle and the dipole moment of the DPPC headgroup [28]. A first notable effect is the disappearance of the characteristic quasi-plateau of DPPC, regardless of temperature, indicating that the presence of particles disrupts the typical phase behavior of the pristine DPPC monolayer. In all cases, the isotherms obtained in the presence of NPs exhibit a monotonic increase of the surface pressure upon compression, consistent with previous observations for DPPC interacting with titanium dioxide particles [17]. Additionally, the collapse surface pressure is systematically lower than that observed for pure DPPC at the same temperature. These findings suggest that nanoparticles introduce packing defects that reduce monolayer stability. Overall, the observed changes indicate that the presence of BT particles in the sub-phase distort lipid packing, in agreement with previous results on the interaction with other types of particles, such as silicon dioxide [15] and titanium dioxide [17].

Another observed effect of the presence of particles in the sub-phase concerns the temperature dependence of the limit area. In fact, contrary to what observed for DPPC spread at the water-air interface, in this case this parameter appreciably increases with temperature. Moreover, while at the lowest temperature it is found below the corresponding value without particles, at the highest temperature (32 °C), the limit area appears appreciably increased by the presence of particles. This behavior suggests the occurrence of an excluded area effect associated with the incorporation of the particles, which becomes more relevant as the temperature increases.

In addition to the temperature dependent modification of the limit area, the presence of BT in the DPPC monolayer affects the whole isotherm in a temperature dependent manner.

As occurred for DPPC monolayers at the bare air/water interface, the isotherms overlap at the lowest compression degrees, i.e., the highest values of the area available per DPPC molecules, independently of temperature. However, as the compression progresses, a clear compression degree dependent behavior is observed which aligns well with the temperature induced expansion expected for lipid monolayers [27]. Going more in detail, one can observe that, independently of the temperature, when the compression degree is small, the interaction with particles reduces slightly the surface pressure. This suggests that the particles interacting with DPPC molecules affect their lateral interaction, and consequently their packing. However, advancing with the compression, the effect of particles on the behavior of DPPC monolayer is different at the different temperatures. At the lowest temperatures (20 °C and 25 °C), the surface pressure remains below the values found for DPPC alone. This can be probably ascribable to the fact that particles penetrate into the lipid film, reducing the effective concentration of DPPC at the interface, as a certain number of molecules of DPPC adsorb onto particle surface, and weaken the lateral interaction between DPPC molecules. This results in a worsening of the lateral packing in the monolayers, which is translated in a reduction of the film rigidity (see Figure 5c,d).

The situation changes when the temperature is increased, and the effect of BT particles on the phase behavior of DPPC passes from a penetration/adsorption-dominated regime to a regime governed by the excluded-area induced by the incorporation of nanoparticles with an increased contribution of the steric-hindrance. This can be understood considering the fluidification of DPPC with temperature (see Figure 4b and Figure 5b). At this point, nanoparticles encounter difficulties integrating into poorly packed films and behave as rigid, non-compressible obstacles. In addition, thermal motion increases their effective cross-section. The combination of these effects results in a sharper, monotonic rise in surface pressure, which is the result of the BT induced disruption of the cooperative lipid ordering, which leads to increased monolayer rigidity (see Figure 5b). Overall, the observed temperature-dependent trend indicates that BT-DPPC interactions remain effective up to near-physiological conditions, supporting the material’s promise for in vivo biomedical imaging where local temperatures approach 37 °C.

#### 2.2.2. Effect of the NP Concentration in the Sub-Phase

**Figure 6:**
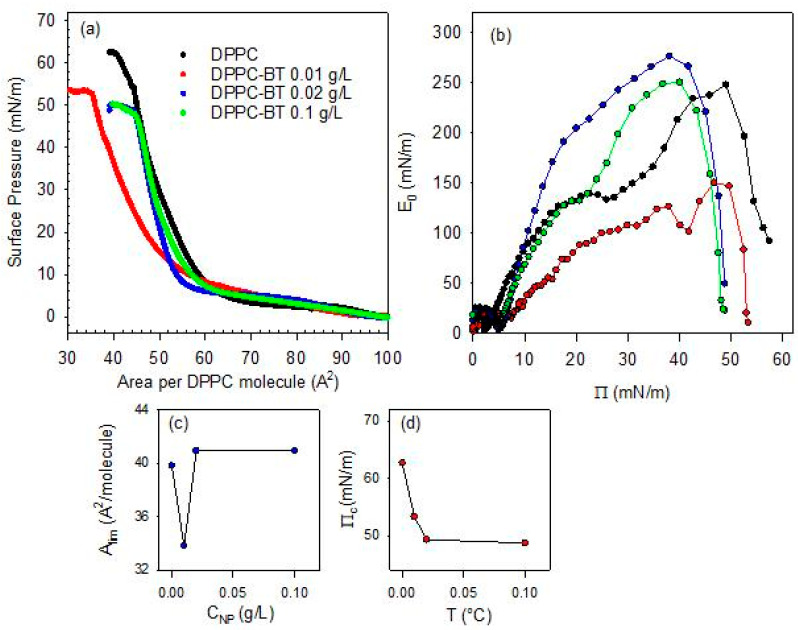
Surface pressure-area per DPPC molecule isotherms for DPPC monolayers spread onto BT aqueous dispersions with different NP concentration (**a**) and associated quasi-equilibrium dilational elasticity versus surface pressure evaluated from the isotherms (**b**), limit area (**c**) and collapse pressure (**d**) versus NP concentration.

The effect of the BT NPs in the sub-phase on the phase behavior of the DPPC monolayer has also been investigated as a function of the NP content. This analysis has been carried out at 20 °C and for NP concentration in the sub-phase, C_NP_, varying within the range 0.01–0.1 g/L. The isotherms obtained are reported in Figure 6, together with that corresponding to bare DPPC, for sake of comparison

From a qualitative point of view, the effect of the increase in particle concentration in the sub-phase is similar to that found as the temperature increases. This underscores the complex interplay between lipid packing and nanoparticle insertion at the air/water interface. At the lowest concentration of the sub-phase (0.01 g/L), there is a dominant role of the depletion of the DPPC molecules from the interface to the particle surface, which reduces the effective density of the monolayer and shifts the isotherm towards lower values of the area available per DPPC molecule. However, as occurred with the increase in temperature, the role of DPPC depletion starts to compete with the steric hindrance associated with the increase in the number of particles that penetrate into the monolayer as their concentration in the sub-phase increases. This shifts the isotherms to higher values of the area available per DPPC molecule, which leads to a situation where particles act as rigid obstacles. When these obstacles enter in contact the surface pressure increases sharply. This differs from the situation occurring at low particle concentration where the adsorbed amount of BT NPs is expected to remain relatively small but enough to provoke an efficient depletion of DPPC from the interface. Similar behavior was reported in [16] for the interaction between DPPC and titanium dioxide nanoparticles at the air/water interface. The above discussed scenario is compatible with the dependence of the A_lim_ on sub-phase concentration (see Figure 6b). Similar concentration-dependent disruptions have been reported for magnetite nanoparticles coated by different polymer shells interacting with DPPC monolayers, where polymer coatings modulate the extent of lipid disorder [29].

In addition to the discussion above, the isotherms corresponding to DPPC monolayers spread onto BT aqueous dispersions exhibit a loss of the quasi-plateau region. This trend parallels the effect of increasing temperature, and confirms the strong distortion induced by particles on the lateral packing and cohesion of DPPC monolayers. On the other hand, this scenario is confirmed by a decrease in the collapse pressure by more than a 10% in relation to that of DPPC monolayers at the bare air/water interface (see Figure 6c).

Further details on the effect of the concentration of the sub-phase on the mechanical stability of DPPC monolayers can be obtained by analyzing the quasi-equilibrium dilational elasticity of the monolayers reported in Figure 6d. As discussed in the previous section, the quasi-equilibrium elasticity decreases significantly with a relative small NPs concentration in the sub-phase (0.01 g/L). This is ascribed to the distortion on the interfacial packing of the DPPC and the consequent weakening of the interfacial cohesion. However, this distortion appears less evident when the concentration of nanoparticles in the sub-phase is increased. In this case, in fact, the quasi-equilibrium elasticity appreciably increases, even reaching values that are higher than those corresponding to the pristine DPPC monolayer at the air/water interface. It is worth noting that at the highest particle concentration evaluated (0.1 g/L), the quasi-equilibrium dilational elasticity drops again, which may be explained by considering that an excessive particle loading may disrupt lipid packing and thereby compromising film integrity. At this point, the role of incorporated particles is essential to understand the mechanical performance of the monolayers, as was previously observed when the effect of the temperature was considered. This behavior is consistent to the findings by Leonardi et al. [30] in a recent study of the nanoparticle-mediated lipid membrane fusion, where subtle physical determinants govern the balance between destabilization and reinforcement of the lipid membrane.

### 2.3. NPs Interaction with BSA

#### 2.3.1. Interaction Between NP and BSA in Bulk

Albumin proteins dissolved in the aqueous phase are expected to adsorb at the surface of NPs modifying some of their properties, like surface charge and/or amphiphilic character, which may have relevant consequences especially for their application as SHG probes in bio-imaging.

**Figure 7:**
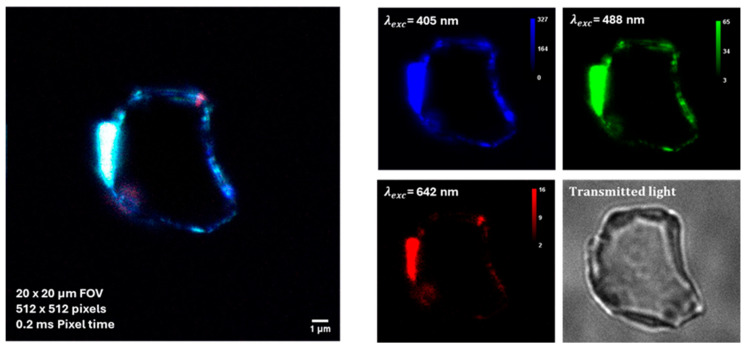
Auto-fluorescence imaging of protein–NPs complexes by super-resolved Image Scanning Microscopy (ISM). BSA concentration corresponds to the physiological concentration in blood, yhat is 50 g/L.

Some preliminary tests on BT dispersions additivated by BSA were performed by using a super-resolved Image Scanning Microscopy (ISM) [21]. As shown in Figure 7, the auto-fluorescence enabled the detection of protein-corona formation in BT dispersions added with BSA at a concentration corresponding to the physiological concentration in blood, that is 50 g/L (about 7.53 × 10^−4^ M).

As albumin proteins are characterized by an anionic character at physiological pH, being the surface charge of BT particle also negative, the observed protein-particle binding can appear counterintuitive. However, it is known from literature [31] that negative charged albumin may adsorb on nanoparticles, forming protein corona, via a combination of interactions which includes hydrophobic forces, hydrogen bonding and van der Waals interactions. In addition, in specific cases like the present one, the protein corona formation may occur as a result of the electrostatic association between the acrylate groups on the surface of BT particles and the positively charged patches existing along the BSA chain [32].

**Figure 8:**
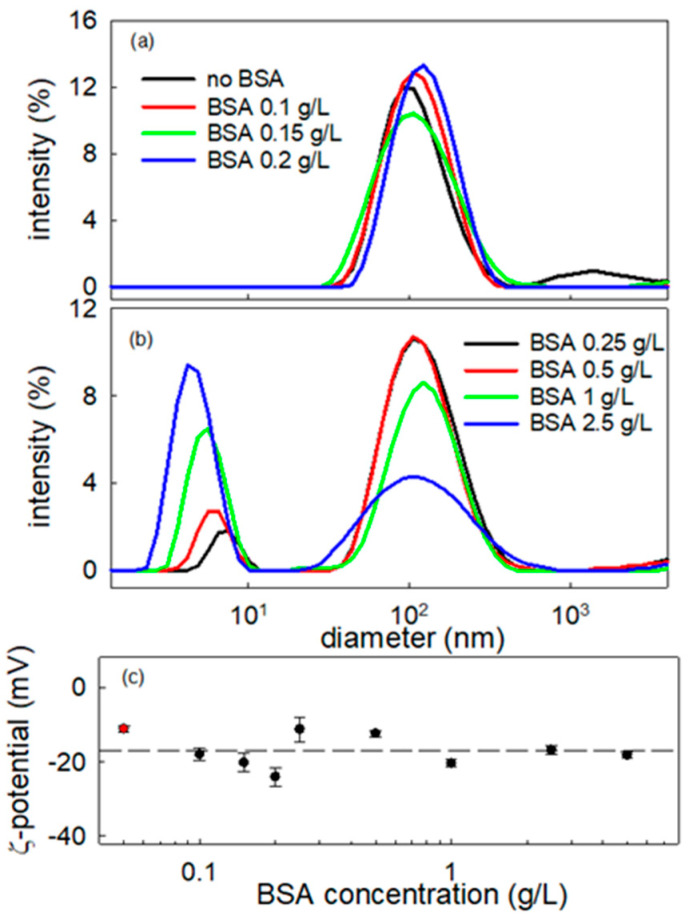
Intensity-weighted particle size distributions of BT-BSA nanometric dispersions for particle concentration at 0.01 g/L and different values of BSA concentration (**a**,**b**), and ζ potential of the same dispersion versus BSA concentration (**c**). The red symbol corresponds to BT NPs dispersion without BSA. Dashed line indicates the average value, i.e., −16.9 mV.

On the basis of these preliminary microscopy observations, a study is here presented aimed at obtaining deeper information on the interaction of BT NPs with BSA, by analyzing bulk properties, like Particle Size Distribution by DLS and zeta potential, ζ. Figure 8 reports the intensity weighted particle size distribution obtained by DLS measurement of BSA-BT composite dispersions at fixed NPs concentration of 0.01 g/L, while varying the BSA content within the range 0.1–2.5 g/L. The corresponding z-potential values are also reported versus the BSA concentration. These latter prove that the adsorption of albumin at the particle surface does not affect significantly the surface charge which keeps its negative value, thereby ensuring a good stability of the dispersions.

The particle size distribution of the BT dispersion without BSA shows a maximum at about 100 nm. Adding BSA to the dispersion the same peak is still present, shifting slightly with the increase in nominal BSA concentration in the dispersion. This is aligned with the formation of a protein corona on the BT particle surface suggested from the ISM images. Further increases in BSA concentration above 0.20 g/L and higher lead to the appearance of a second peak, centered around 4–5 nm, in the diameter distribution. This may be expected to correspond to BSA free molecules [32]. which according to previous report in the literature present a hydrodynamic diameter in the range 4–7 nm depending on the specific conditions of the solution [33,34,35]. Thus, from these results we can deduce that the maximum coverage of the NP is obtained at a BSA concentration within 0.2–0.25 g/L range, where the presence of free BSA molecules starts to be detected. Moreover, from the relative intensity of the two peaks appearing in the size distribution, it can be assumed that once the maximum coverage of BT nanoparticles is reached, the excess of BSA remains solubilized in the aqueous medium. This is clear by the significant increase in the intensity corresponding to the scatterers with the smallest size as the BSA concentration increases (notice that the scattering intensity depends on the six power of the size). In addition, the modest shift in the BT peak position reflects monolayer protein corona formation (BSA $R_{H}$ ≈ 3–3.5 nm), confirmed by unchanged negative zeta potential and saturation at ~0.25 g/L BSA. Larger shifts or zeta neutralization would indicate protein multilayer adsorption or particle aggregation, which are absent here.

#### 2.3.2. Adsorption of BSA and BT-BSA Complexes

The amphiphilic character of albumin is well known and many studies can be found in literature dealing with the adsorption properties of BSA at water-air interfaces [36,37,38]. For this reason, the BT–BSA complexes investigated in the previous section are expected to be characterized by a certain degree of amphiphilicity.

**Figure 9:**
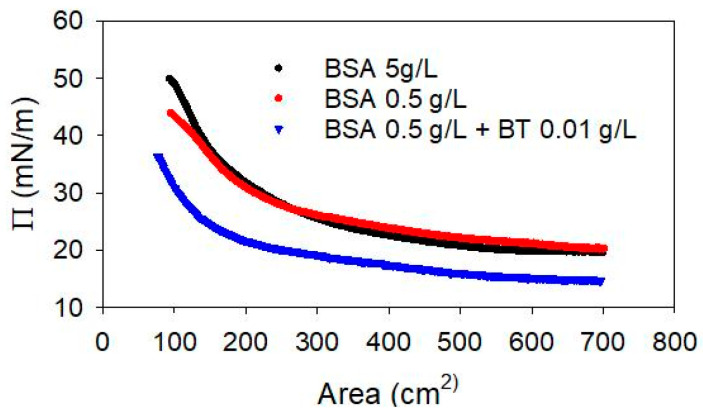
Compression isotherm, at T = 25 °C, of the adsorbed layer of BSA with and without BT NPs in the sub-phase.

To verify this characteristic of the complexes, the compression isotherm of the BSA adsorbed layer, with and without the presence of BT NPs in the sub-phase, has been investigated and the results are reported in Figure 9. The experiments have been carried out, in particular, for 5 g/L and 0.5 g/L of BSA, in the absence of NPs, and for a system containing BT NPs at 0.01 g/L with BSA at 0.5 g/L, corresponding to 7.5 × 10^−6^ M.

The results show similar features for the two BSA solutions without NPs. Moreover, the surface pressure at adsorption equilibrium, before the compression, are very similar, i. e., 20.1 mN/m and 19.6 mN/m, at 0.5 g/L and 5 g/L, respectively, in agreement with previous findings on the adsorption isotherm of BSA at the water-air interface [39,40]. In the presence of BT NPs, the equilibrium surface pressure is found to be 14.6 mN/m, lower than that found for BSA alone, while the feature of the compression isotherm does not differ in an appreciable way. The reduction of the equilibrium surface pressure, which is the most evident effect of the presence of NPs in the sub-phase, can be explained considering the capture of BSA molecules by the NPs and the consequent reduction of the adsorption of free amphiphilic molecules.

In addition, based on the DLS results reported above, it is possible to estimate the composition of the mixed dispersion here investigated, that is the free BSA amount is likely between 0.25 and 0.3 g/L (approximately 4 × 10^−6^ M) which, according to literature data on the adsorption properties of BSA [37,41], corresponds to an equilibrium surface pressure of about 15 mN/m, which is in good agreement with the value found here in the presence of NPs. Thus, the results obtained provide further evidence for the formation of BSA-NP complexes, while the adsorption of these complexes at the water-air interface is not directly proven.

### 2.4. Interaction of BT-BSA Complexes with DPPC Monolayer

To investigate in more detail, the interaction of the BT-BSA complexes with the DPPC monolayer and their potential incorporation into the layer, an experimental procedure has been adopted according to that proposed in ref. [42]. The experiment utilises a spread DPPC monolayer in the Langmuir balance and consists in the introduction of a certain amount of dispersion into the aqueous sub-phase once fixed surface pressure values are reached, while continuously monitoring the surface pressure at a constant surface area, i.e., the surface density of lipid. For the experiments reported here, 5 mL of concentrated dispersion was introduced, with a composition appropriate to obtain a final sub-phase composition equivalent to that used in the studies described in the previous section, that is 0.01 g/L of NPs and 0.5 g/L of BSA.

**Figure 10:**
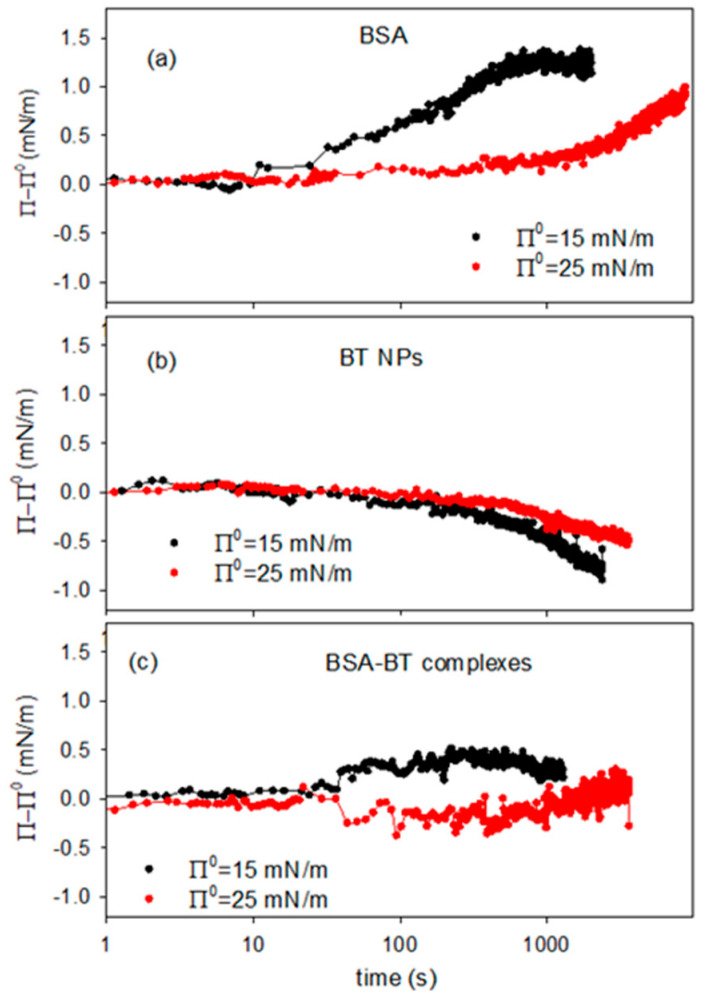
Variation of the surface pressure of DPPC monolayer after the reaching of an equilibrium surface pressure, Π^0^, and the introduction of 5mL of concentrated dispersion containing BSA (**a**), BT NPs (**b**) and BT-BSA complexes (**c**), for different values of reference surface pressure, at T = 25 °C.

Figure 10 reports the variation of the surface pressure, Π–Π_0_, versus time acquired after the introduction on the sub-phase of the concentrated dispersion at two different values of the reference equilibrium surface pressure, Π_0_, of the DPPC monolayer. For sake of comparison, the experiments were performed also for solutions of BSA and dispersion containing BT alone, without protein (Figure 10a,b).

In the case of BSA alone the results demonstrate the adsorption/incorporation of the protein into the DPPC monolayer with the consequent effect of increasing the surface pressure. For BT dispersions, on the contrary, the pressure variation has a negative trend which can be interpreted considering that the incorporation of particles distorts the packing of the monolayer, as discussed in previous section. In fact, the data reported in Figure 10b are coherent with the results on the effect of particle on the compression isotherm reported in Section 2.2 (Figure 4c).

For BT-BSA composite dispersions (Figure 10c), the surface pressure tends to slightly increase. However, being such variation very low, that is below 1 mN/m, a definitive conclusion on the incorporation of the complexes into the DPPC monolayer is hardly to be obtained. What can be deduced from these results is the fact that the interaction of the BT-BSA complexes with the DPPC monolayer does not imply a relevant effect on its phase behavior, at least under the temperature and sub-phase concentration conditions employed.

Moreover, the lower effect of the BT-BSA complexes on the surface pressure, compared with that of BSA alone, provides additional evidence for the formation of BT-BSA complexes and indicates that this association provokes a reduction of the effective BSA concentration. On the other hand, the small ΔΠ (<1 mN/m) reflects reduced amphiphilicity of BT-BSA complexes vs. free BSA, consistent with protein corona formation sterically limiting interfacial adsorption. This effect is statistically significant (Wilhelmy plate resolution ~0.1 mN/m) and reproducible.

## 3. Materials and Methods

### 3.1. Materials

The products utilized for the synthesis of BT particles were: BaCl_2_·2H_2_O (Merck, Darmstadt Germany, 99.9%), NaOH pellets (anhydrous) by Merck (Darmstadt, Germany), ≥98%, aqueous solution of TiCl_4_ by Acros (Geel, Belgium), 99.9%. For the dispersion stabilization, aqueous solution of polyacrylic acid with average molecular weight of 2 kDa by Acros Chimica (Milan, Italy) was used.

1,2-Dipalmitoyl-sn-glycerol-3-phosphocholine (DPPC) was purchased from Merck (Darmstadt, Germany) at 99% purity and used without further purification. The molecular weight of this lipid is 734.1 g/mol. Solutions of lipids for the spreading were prepared using chloroform for HPLC from Merck (Darmstadt, Germany).

Bovine Serum Albumin, BSA, was purchased by Merck (Darmstadt, Germany). The molecular weight of this BSA is around 66.4 kDa, and its physiological concentration in human serum is ~50 g/L (corresponding to about 7.53 × 10^−4^ M) [34].

Water used for the preparation of all solutions and dispersions was purified by a multicartridge system, Elix plus Milli-Q, Millipore (Darmstad, Germany) providing a resistivity greater than 18 MΩ·cm and a surface tension of 72.5 mN/m without any appreciable kinetics over several hours.

### 3.2. Methods

#### 3.2.1. Synthesis and Functionalization of BT Particles

The synthesis of BT particles was conducted using a hydrothermal process. Precipitation of crystalline BT can be performed in a strong alkaline solution containing sodium hydroxide according to the overall reaction:

BaCl_2_(aq) + TiCl_4_(aq) + 4NaOH(aq) → BaTiO_3_(s) + 4NaCl(aq) + 3H_2_O(l)

where (aq) denotes a salt dissolved in aqueous solution. When TiCI4 is dissolved in water, it undergoes hydrolysis, forming Ti(IV) hydroxo complexes or Ti(IV) polyanionic species, depending on the pH and the concentration of the solution.

The synthesis proceeds in two sequential stages. First, a gelatinous suspension of amorphous, hydrated titanium dioxide is produced through rapid hydrolysis of a BaCl_2_/TiOCl_2_ solution by adding a concentrated solution of NaOH. In the second stage, this gel reacts with Ba^2+^ ions remaining in solution to form crystalline BT particles. The reaction was carried out at elevated temperatures (80–100 °C) under high pH (pH ≈ 14), which promote the formation of BaTiO_3_. By adjusting processing parameters such as temperature and ionic concentrations the resulting particle size can be tailored from approximately 10–20 nm up to more than 1 µm.

After synthesis, the precipitate was washed several times with distilled water by centrifugation in order to remove residual Na^+^ and Cl^−^ ions and then left moist to prevent NPs agglomeration during the drying process. The wet powder was diluted to a known volume with distilled water and then titrated by gravimetric analysis, by drying three aliquots and weighing the resulting product, in order to define the starting concentration of the prepared suspension.

With the aim of stabilizing BT aqueous dispersions, appropriate agents were added which, by adsorbing on the solid surface of the NPs, increase their colloidal stability modifying the surface charge. These additional components, beside to be effective for the optimization of the Drop Size Distribution of NPs in bulk phase, must satisfy biocompatibility requirements to be appropriate for utilization in biomedical applications. After the synthesis, polyacrylic acid (PAA) was introduced to enhance dispersion stability, and the optimization of the PAA/BT surface-coverage ratio ensured effective stabilization through surface charge modulation and polymer adsorption, without significantly altering the aqueous phase composition.

Concerning the nanoparticle surface functionalization, other additives are under study, like Silanes (i.e., 3-aminopropyltriethoxysilane, APTES) and Phosphonate (i.e., adenosine-5′-triphosphate, ATP), which are expected to improve the colloidal stability and, at the same time, the compatibility in aqueous and biological environments [18]. Moreover, these additives are known from the literature to be used in theranostic applications such as cancer imaging and drug delivery [43,44].

#### 3.2.2. Interaction with Spread and Adsorbed Layers

Lipid monolayers were prepared by spreadng defined aliquots of their chloroform solutions onto the aqueous sub-phase (Milli-Q water or BT aqueous dispersion) contained on a Teflon Langmuir trough using a Hamilton micro-syringe. Knowing both the spread volume and the solute concentration (~1 mg/mL) allowed precise control over the amount of material remaining at the air–water interface once the solvent had fully evaporated. All measurements were performed with temperature controlled with a precision of ± 0.1 °C.

Two different Langmuir balances, equipped with two Delrin barriers allowing symmetric compression/expansion of the free liquid surface, were used for the experiments. A Langmuir trough Nima model 702 by Nima Technologies (Coventry, UK) with a total surface are of 700 cm^2^, and a Langmuir trough KSV Minitrough by KSV Instrument (Finland) with a total working surface area of 243 cm^2^. Surface tension, γ, was monitored using a Wilhelmy plate made from Whatman CHR1 chromatography paper (effective perimeter 20.6 mm), ensuring a zero-contact angle. The surface pressure was calculated as γ = γ_w_ − γ, where γ_w_ corresponds to the surface tension of pure water. Π-A isotherms were recorded by compressing the monolayer at a constant rate of 3 cm^2^/min, a speed previously verified to minimize non-equilibrium artifacts. Compression began one hour after spreading the lipid solution, a waiting period confirmed to be sufficient for complete solvent evaporation and, when nanoparticles were present, for the system to reach equilibrium through nanoparticle–lipid interactions.

The Langmuir trough Nima model 702 by Nima Technologies (Coventry, UK) was also used to evaluate the insertion of BT, BSA, and BT-BSA complexes into lipid monolayers. For this purpose, DPPC monolayers were prepared at the air/water interface and compressed to fixed surface pressures. Once the interface reached equilibrium, a concentrated solution of BSA or concentrated dispersions of BT or BT-BSA complexes was injected into the sub-phase to achieve a nominal concentration comparable to that used in the other experiments. The subsequent time evolution of the surface pressure was then monitored. The compression isotherm of adsorbed layers of BSA and BT-BSA complexes at the air/water interface was also evaluated with the Langmuir trough Nima model 702 by Nima Technologies (Coventry, UK). For this purpose, the adsorption of the BSA or the BT-BSA mixtures was studied until equilibrium is reached, evidenced by the existence of a variation of surface pressure below 0.1 mN/m for a period of 30 min, and then the obtained film was compressed until the minimum area available at a constant rate of 3 cm^2^/min.

#### 3.2.3. Bulk Analysis of the Dispersions

The overall charge of the BT particles and BT-BSA complexes was assessed by measuring their electrophoretic mobility (*u*_e_) using Laser Doppler Velocimetry with a Zetasizer Nano ZS by Malvern Instruments Ltd. (Malvern, UK). The corresponding zeta potential (ζ) was obtained from the measured mobility through Henry’s equation,

(2) $$u_{e}=\frac{2\varepsilon \zeta f\left(\kappa a\right)}{3\eta }$$

In this expression, *ε* denotes the dielectric permittivity of the medium, *η* its viscosity, and f(κa) the Henry function. For the conditions of this study, f(κa) was assigned a value of 1.5, following the Smoluchowski approximation, which is appropriate when the particle radius is large compared with the thickness of the electrical double layer [45].

Dynamic light scattering (DLS) measurements were carried out in a quasi backscattering setup (scattering angle θ = 173°) using a Zetasizer Nano ZS (Malvern Instruments Ltd., Malvern, UK). The instrument employs the red emission line of a He–Ne laser with a wavelength of 632 nm. Under these conditions, DLS provides the apparent diffusion coefficient, D_app_, of the dispersed particles at 25 °C, assuming their motion is governed by Brownian dynamics. The corresponding apparent hydrodynamic diameter, d was calculated from D_app_ using the Stokes–Einstein relation:

(3) $$d=\frac{k_{B}T}{3\pi \eta D_{\mathrm{app}}},$$

where k_B_ is the Boltzmann constant, T the absolute temperature, and η the viscosity of the surrounding medium. It is important to emphasize that DLS analysis requires optically transparent dispersions to minimize the influence of multiple scattering [46]. Size distributions were obtained using intensity-weighted analysis (standard for polydisperse NP dispersions), reporting hydrodynamic diameter distributions from correlation function analysis. Polydispersity Index (PDI) values were not calculated due to the presence of scatterers with different characteristic dimensions, which violate Gaussian distribution assumptions required for meaningful PDI reporting. Instead, size distribution profiles (Figure 8a,b) provide complete characterization of the polydisperse systems.

#### 3.2.4. Microscopy, SHG Testing and SEM Analysis

The evaluation at micrometric scale of the particle sizes in BT dispersion, has been performed using a DVM6-M optical microscope, Leica microsystems GmbH (Hamburg, Germany).

For SHG and auto-florescence tests a custom optical microscope based on a 25-element SPAD detector array and FPGA-based acquisition electronics detection unit was adopted. This device is able to combine SHG imaging and super-resolution Image Scanning Microscopy (ISM) [21]. In this work, the formation of protein-corona around BT NPs was detected using this label-free optical microscopy method. Unlike conventional fluorescence microscopy, ISM provides a higher resolution, allowing the detection of protein-NP interactions at a near-molecular level. The intrinsic auto-fluorescence of the protein-NP complexes was used to visualize the protein-corona, without the need for additional fluorescent tagging. This approach avoids altering the native properties of the proteins and NPs. The microscope setup adopted for the present study was characteruzed by an excitation wavelengths of 1.150 nm (for SHG imaging) and 405 nm, 488 nm, and 642 nm (for ISM imaging). The field of view (FOV) was 20 × 20/40 × 40 µm with a pixel resolution of 512 × 512 and the pixel dwell time: 0.2 ms.

The morphology of the BT powders was characterized by scanning electron microscopy using SEM, LEO 1450VP by LEO Electron Microscopy Ltd. (Cambridge, UK).

## 4. Conclusions

Barium titanate (BT) particles were synthesized with precise control over morphology and size and formulated into aqueous dispersions exhibiting reproducible colloidal behavior. Stabilization was achieved using polyacrylic acid (PAA), whose loading was optimized according to the measured specific surface area and the adopted preparation route to balance dispersion stability with minimal alteration of the aqueous phase. The resulting suspensions display two distinct populations: micron-scale aggregates visible by optical microscopy and a nanoscale fraction, centered near 100 nm determined by DLS after centrifugation of the pristine dispersion. This latter fraction is composed of aggregates of primary particles with diameters of 15–20 nm.

Interactions between BT nanoparticles and biological components were explored both in bulk and at model interfaces. In the bulk phase, bovine serum albumin (BSA) readily associates with the particles, producing stable complexes and clearly detectable protein coronas by super-resolution microscopy. At the air/water interface, incorporation of BT into DPPC Langmuir monolayers alters the monolayer phase behavior in a manner that depends on temperature and nanoparticle concentration: at lower temperatures and concentrations, particle penetration and adsorption reduce effective lipid packing and film rigidity, whereas at higher temperatures or loadings steric exclusion and obstacle-like behavior dominate, producing sharper increases in surface pressure and modified mechanical responses. The temperature-dependent interactions observed across 20–32 °C, approaching physiological conditions (~37 °C), demonstrate controlled BT nanoparticle incorporation into lipid monolayers without compromising interfacial stability. This behavior has direct implications for SHG nanoprobes operating in physiological environments (37 °C), where protein corona formation and membrane interactions govern targeting, bio-distribution, and biocompatibility.

Taken together, these findings demonstrate that SHG-active BT nanoparticles can be produced with controlled size and surface chemistry and that their interactions with proteins and lipid films are governed by a balance of adsorption, electrostatic and steric effects. Such behavior has direct implications for the design of SHG probes for biomedical imaging and for nanoparticle-based delivery systems, where protein corona formation and membrane interactions will influence targeting, bio-distribution and biocompatibility. This study contributes to a broader program aimed at developing efficient, biocompatible SHG nano-probes. Ongoing work will concentrate on tuning nanoparticle–lipid affinity, refining surface functionalization to modulate corona formation, and evaluating performance in progressively more complex biological media and model membranes to bridge toward in vitro and in vivo validation.

As a concluding remark, it is important to underline that the non-linear optical properties of the BT particles investigated make them particularly promising for optical bio-imaging, where it is important a high-contrast detection under conditions compatible with deep-tissue excitation, as previously emphasized by Bonacina et al. [2] and to open up new possibilities for their use in real-time imaging, diagnostics and, possibly, therapeutic monitoring, aligning with recent advances in nano-medicine, as outlined by Soos et al. [3]. Moreover, the observation that BT particles can be visualized without fluorescent labels highlights the intrinsic advantage of SHG-active materials, enabling their detection and monitoring directly at the interface while eliminating the artefacts and limitations typically introduced by conventional fluorophores.

## Data Availability

The raw data supporting the conclusions of this article will be made available by the authors on request.
